# Supplementary material for: Association between oral microbiome and seven types of cancers in East Asian population: a two-sample Mendelian randomization analysis
Source: Front Mol Biosci. 2023 Nov 21;10:1327893. doi: 10.3389/fmolb.2023.1327893 (PMC10702768; doi:10.3389/fmolb.2023.1327893)
Supplement: Supplementary file 1 [file DataSheet1.ZIP › Supplementary Materials/MR plots of saliva/saliva═╝/colorectal cancer/pheno.2027_to_colorectal cancer_forest.pdf]

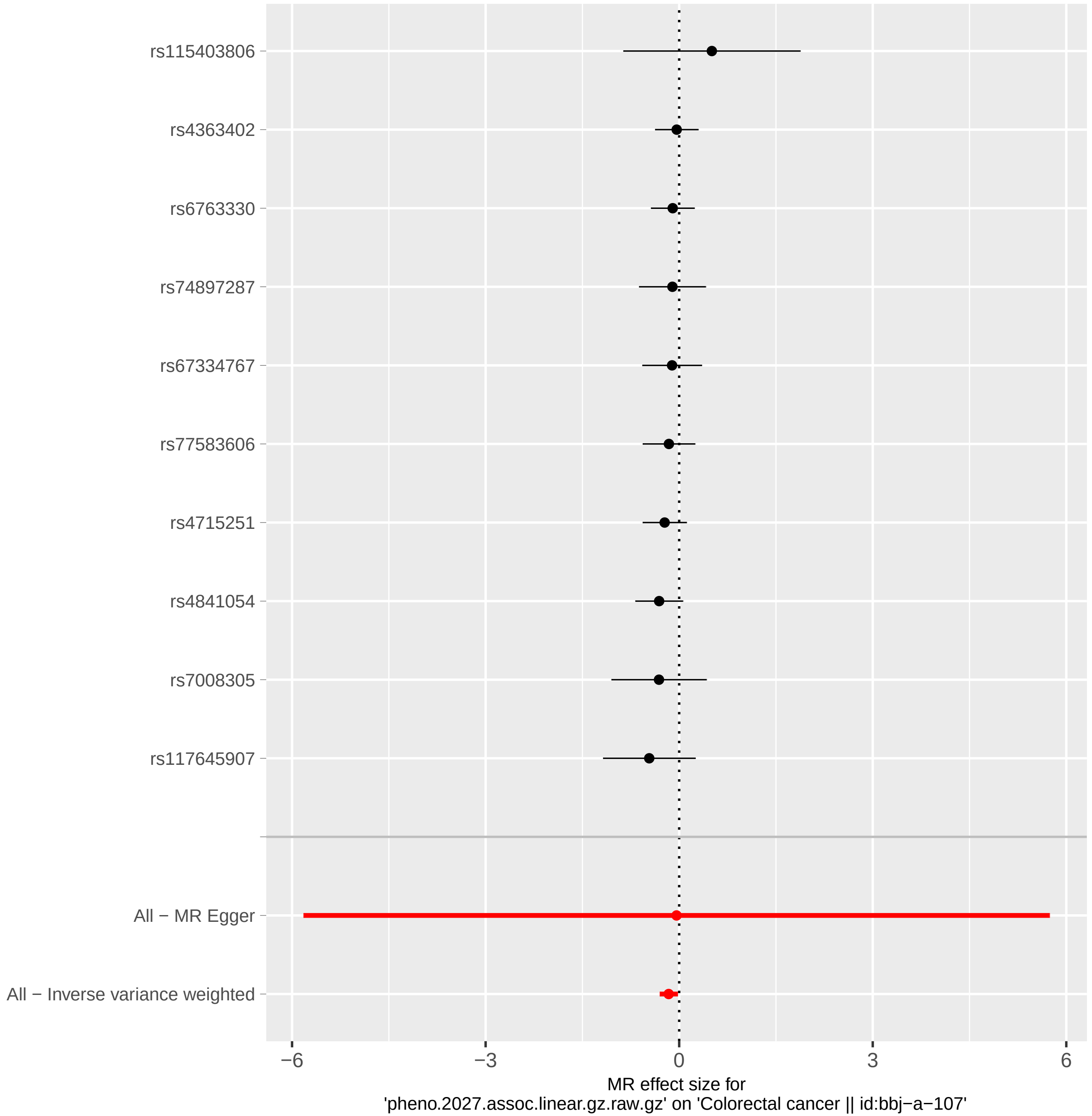

rs115403806
rs4363402
rs6763330
rs74897287
rs67334767
rs77583606
rs4715251
rs4841054
rs7008305
rs117645907
All − MR Egger
All − Inverse variance weighted
−6
−3
0
3
6
MR effect size for
'pheno.2027.assoc.linear.gz.raw.gz' on 'Colorectal cancer || id:bbj−a−107'
